# Fixation of Flowable Composite Resin Within Crossing Sutures for the Closure of Oroantral Communications: Two Clinical Cases and Literature Review

**DOI:** 10.1155/carm/9968600

**Published:** 2025-06-26

**Authors:** Aibek A. Sissenaliyev, Madina A. Kurmanalina, Aruzhan M. Aitmukhanbetova

**Affiliations:** ^1^AS Dental Clinic, Atyrau Region, Kurmangazy Village, Kazakhstan; ^2^Department of Dentistry and Maxillofacial Surgery, West Kazakhstan Marat Ospanov Medical University, Aktobe, Kazakhstan

**Keywords:** case report, flowable composite resin, maxillary sinus, oroantral communication, tooth extraction

## Abstract

**Background:** Oroantral communications (OACs) are pathological openings between the oral cavity and the maxillary sinus, often resulting from dental extractions. If not treated promptly, they may lead to sinus infections and persistent oroantral fistulas (OAFs).

**Objective:** This report aims to present a minimally invasive technique for the closure of OACs using flowable composite resin stabilized within a network of crossing sutures and to assess its clinical efficacy.

**Case Descriptions:** Two male patients, aged 27 and 31, presented with OACs measuring 8 mm and 6 mm in diameter following maxillary molar extractions. Both cases were managed within 24–48 h postextraction.

**Intervention:** Under local anesthesia, the extraction sockets were sutured using absorbable threads, creating a cross-matrix over the alveolus. Flowable composite resin was then applied into and over this suture network to form a sealing layer. The material was light-cured, and a secondary application was performed following socket curettage to promote stable blood clot formation. In this technique, the flowable composite is mechanically stabilized within the network of crossing sutures, which enhances the retention of the material, ensures effective sealing of the OAC, and supports a more predictable and secured healing process.

**Outcomes:** In both cases, healing was uneventful, with complete closure of the OAC and no signs of sinusitis or fistula formation. Follow-up at 8 and 9 months confirmed successful mucosal healing and radiographic bone regeneration.

**Conclusion:** The fixation of flowable composite within a suture matrix offers a novel, cost-effective, and reproducible technique for the immediate closure of small-to-moderate OACs. This approach enhances mechanical stability, improves sealing, and facilitates predictable healing while avoiding more invasive surgical interventions.

## 1. Introduction

Oroantral communications (OAC) are characterized by an abnormal connection between the oral cavity and the maxillary sinus, which arises from the loss of the soft and hard tissues that usually separate these two areas. OAC can result from various causes including dental infections [1], radiation therapy [2], consequences of removing maxillary cysts and tumors [3], osteomyelitis [4], and trauma [5]. Defects with a width of less than 2 mm, devoid of epithelialization, have the potential for spontaneous resolution in the absence of infectious agents, a process facilitated by the formation of a primary blood clot [6]. Conversely, when the defect is larger, the probability of spontaneous recovery diminishes, and the risk of infection escalates due to exposure to the oral microbial flora, potentially resulting in sinusitis [7]. Osseous defects exceeding 2 mm necessitate targeted surgical interventions to close the communication effectively and forestall the development of sinus infections. The timing of such interventions is a crucial prognostic factor; the existing literature supports a success rate of 90–95% in OAC when treatment is initiated within the first 24–48 h. Delays beyond 48 h significantly increase the likelihood of subsequent sinus infections and the formation of oroantral fistulas (OAFs) [8].

Surgical techniques for the closure of OAC encompass a variety of methods. These include gingival suturing [9], the use of connective tissue grafts and flaps [6], hemostatic sponge and foams [10], and even autotransplantation of third molar [11]. Common soft tissue grafts employed are palatal rotational flaps, palatal transposition flaps, buccal advancement flaps, and combinations of buccal and palatal flaps. Despite its widespread use, the buccal advancement flap has some notable drawbacks. One major concern is the potential for a considerable reduction in the vestibule due to the upward shift of the mucogingival line. This alteration often requires subsequent vestibuloplasty to expand the area of keratinized tissue. Additionally, complications such as buccal edema and postoperative pain are frequently reported with this technique [8].

The palatal rotated flap is another surgical approach for closing OACs. This method involves creating a full-thickness flap that includes the palatal artery, ensuring good vascular supply to the flap. However, a significant limitation of this technique is the restricted mobility of the flap, which compels the surgeon to create a large defect in the palatal area. This often results in extensive scarring. Furthermore, donor site morbidity is common with this method, and patients frequently experience considerable postoperative pain due to the procedure [12].

Minimally invasive approaches to managing OAC involve the use of autogenous grafts such as platelet-rich fibrin (PRF) and various synthetic bone graft materials. These synthetic options include materials such as gold foil, aluminum, titanium, tantalum, and polymethylmethacrylate plates [6]. These materials are chosen for their biocompatibility and structural properties, which support the stabilization and healing of the affected area without requiring invasive surgical intervention.

Flowable composite resin is widely utilized in restorative dentistry, particularly as liners and bases for the restoration and repair of minor dental defects. Interestingly, the concept of using composite to close an OAC bears some resemblance to techniques involving metal plates. However, due to the greater accessibility of flowable composite in dental practice, this material has shown to be an effective alternative. Consequently, the aim of this article is to present the positive outcomes achieved through our method of using flowable composite resin for OAC closure.

## 2. Materials and Methods

### 2.1. Case Report #1

A 27-year-old male patient sought consultation and treatment at the Dental Clinic of Atyrau, Kazakhstan. The patient experienced pain in the left maxilla, specifically around the first molar, identified as Tooth 26 according to the Federation Dentaire Internationale (FDI) classification and reported having a toothache that was aggravated by biting on the tooth.

**Figure 1:**
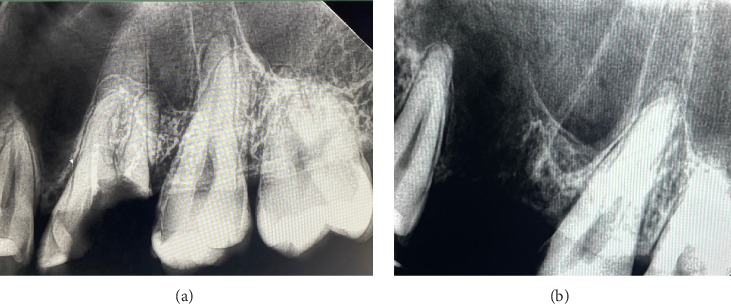
Periapical radiograph of Tooth 26: (a) before the extraction and (b) after the extraction.

The patient in good general health with no systemic diseases or allergies, and nonsmoker underwent a series of diagnostic tests including extraoral, intraoral, and radiological examinations. The extraoral examination did not reveal any abnormalities. During the intraoral examination, an increased sensitivity to vertical percussion was noted on Tooth 26, although the mucosa surrounding the tooth showed no changes. Periapical radiography was conducted using the Planmeca ProX intraoral X-ray unit, while orthopantomography (OPG) was carried out with the Planmeca ProMax 3D Plus, utilizing Planmeca Romexis imaging software for both procedures. The periapical radiographs showed that the roots of the involved teeth were closely situated to maxillary sinus (Figure 1(a)).

The patient provided informed written consent for the procedures as well as for the use of their data and photos for publication purposes.

**Figure 2:**
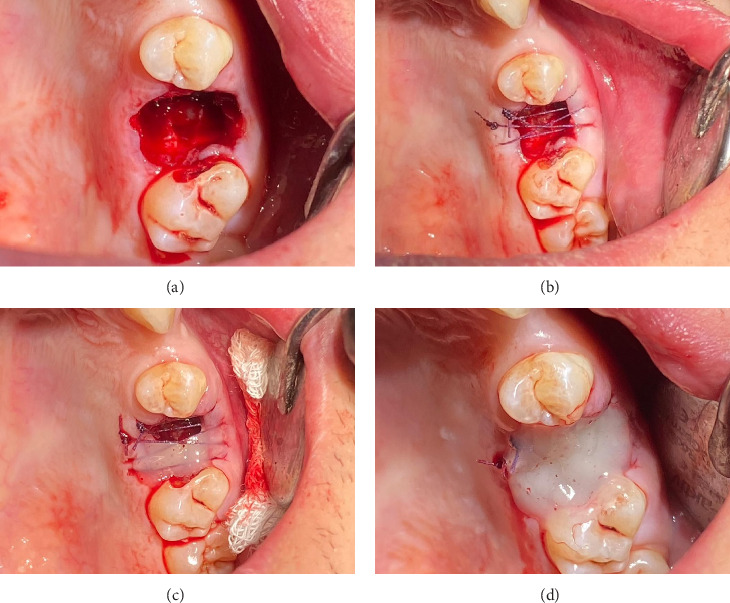
Intraoperative photograph. (a) Communication between the oral cavity and the left maxillary sinus through the extraction socket has been observed. (b) Socket of the extracted tooth was sutured. (c) Small gap was maintained between the composite layer and the socket of the extracted tooth to perform curettage in the socket to facilitate the formation of a blood clot. (d) Final intraoral view, the socket is isolated from the oral environment.

The surgical procedure, illustrated in Figure 2, was carried out under local anesthesia administered through an injection. The posterior superior alveolar and palatal nerves were anesthetized using 2.4 mL of 4% articaine hydrochloride with 1:200,000 epinephrine (2 ampules; Articaine Inibsa 1:200,000, Spain). Tooth 26 was extracted in an atypical technique without the use of dental forceps. The tooth was sectioned using a round bur attached to utilizing the CX207-F 45° LED surgical handpiece (Coxo, Guangdong, China) with copious irrigation (0.05% chlorhexidine). Following crown-root separation, the roots were delicately removed using the straight elevator (Truewin, Pakistan). An OAC was confirmed using a mechanical assessment with an alveolar spoon, supplemented by performing nose and mouth blowing (Figure 2(a)), and further substantiated through radiological verification (Figure 1(b)). The maxillary sinus perforation stemmed from the palatine root of Tooth 26. The defect measured approximately 8 mm in diameter.

Prior to suturing, the flaps were mobilized at a 45-degree angle by dissecting the gingiva from the periosteum on the palatal and buccal sides of the socket to a depth of 0.5 cm using a scalpel. Vicryl absorbable sutures (Ethicon, USA) of sizes 4/0 were utilized (Figure 2(b)). The needle was inserted 0.5 cm from the wound margins, with the wound edges brought as close together as possible. Hemostasis was successfully achieved. Subsequently, the network of sutures was covered with a flowable composite resin EsFlow (Spident, South Korea) applied both beneath and over the wound edges and cured. A small gap was maintained between the composite layer and the socket of the extracted tooth (Figure 2(b)). A curettage spoon was then used to gently perform curettage in the socket to facilitate the formation of a blood clot (Figure2(c)). Finally, an additional layer of flowable composite was promptly applied over this and cured using light, effectively creating a cap-like or densely sealed protective layer. This completely isolated the socket of the extracted tooth from the oral environment (Figure 2(d)).

**Figure 3:**
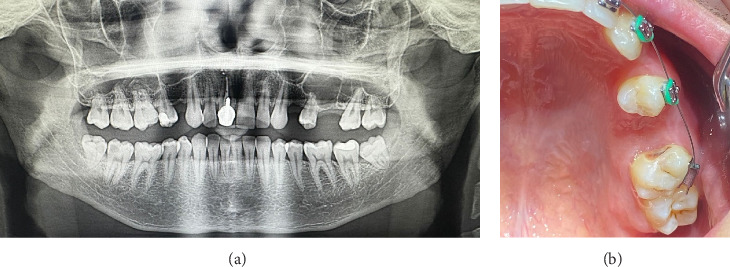
Nine months postsurgery: (a) OPG and (b) intraoral photograph following the placement of braces.

The patient was prescribed antibiotics (Ciprolet 500 mg, every 12 h for 5 days) and probiotics (Linex forte 60 mg, once daily for 5 days). Additionally, decongestants (two drops of 0.1% Naphthyzinum once daily for 5 days) were to be administered into the left nostril. It was also recommended that the patient rinse their mouth with a 0.9% NaCl solution twice daily throughout the 14-day perioperative period. He was also advised to refrain from activities that could increase intraoral or intranasal pressure, such as smoking, using straws for drinking, and actions such as blowing the nose or sneezing. The patient returned for follow-up visits after 24 h and again after 14 days. On the 14th day, both the sutures and the composite resin layer were removed. Nine months following the procedure, the patient attended a follow-up appointment. The patient reported no issues with the oral cavity or sinuses. The intraoral examination confirmed that the soft tissue had completely healed. OPG and photographic images were captured (see Figure 3). Radiographic analysis showed successful bone healing at the surgical site, with no alterations or thickening in the mucosal lining of the maxillary sinus floor.

### 2.2. Case Report #2

A 31-year-old male patient in overall good health was referred to Dental Clinic. He reported experiencing severe pain in the left maxilla around the second molar, designated as Tooth 27. He described the toothache as worsening when biting down on the tooth.

**Figure 4:**
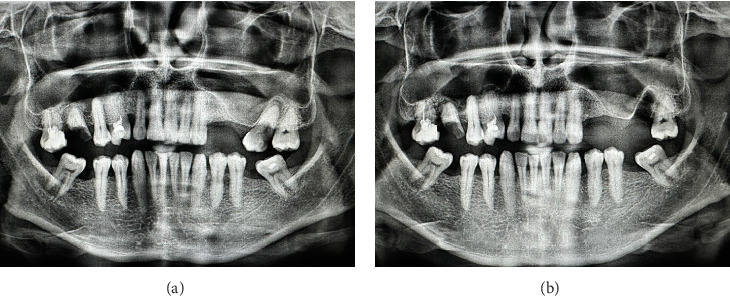
OPG. (a) Tooth 27, before the extraction. (b) After the extraction.

The extraoral examination showed no notable findings. In the intraoral examination, Tooth 27 exhibited increased sensitivity to vertical percussion testing, though the surrounding mucosa showed no visible changes. The OPG demonstrated that the roots of the affected teeth were closely positioned near the maxillary sinus (Figure 4(a)).

The patient gave informed written consent for both the procedures and the use of their data and photos for publication purposes. The procedure for extracting Tooth 27 followed the same protocol as outlined in the first clinical case. The presence of OAC was confirmed through nose and mouth blowing tests, as well as by OPG. Perforation of the maxillary sinus originated from the palatine root of Tooth 27. The diameter of the defect was approximately 6 mm (Figure 4(b)).

**Figure 5:**
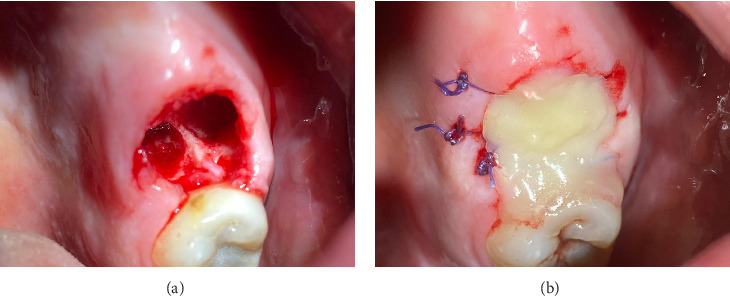
Intraoperative photograph. (a) Communication between the oral cavity and the left maxillary sinus through the extraction socket has been observed. (b) Final intraoral view, the socket is completely isolated from the oral environment.

The suturing of the extraction socket and the application of composite are depicted in Figures5(a) and 5(b).

**Figure 6:**
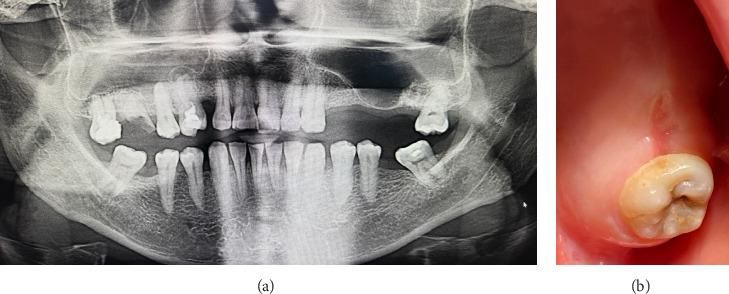
Eight months postsurgery: (a) OPG and (b) intraoral photograph.

The same prescriptions for antibiotics, probiotics, and mouth rinse were issued as in the first clinical presentation. The observation period was set at 24 h and 14 days. Eight months postsurgery, neither maxillary sinusitis nor fistulae were detected in OPG (Figures 6(a) and 6(b)). Additionally, immature bone formation was observed in the extracted tooth socket.

## 3. Discussion

Normal socket healing relies on the formation of a stable blood clot, which supports granulation tissue formation, epithelial migration, and eventual bone regeneration. In the presence of an OAC, this process is significantly disrupted due to the loss of separation between the oral cavity and the maxillary sinus. The entry of air, pressure fluctuations, and microbial contamination impair clot stability and delay healing. This creates a pathological environment that prevents spontaneous closure and promotes chronicity [8, 9]. If left untreated or inadequately managed, OACs may epithelialize and evolve into persistent OAFs. These are frequently associated with chronic maxillary sinusitis [13], nasal regurgitation of fluids [14], and malodor [15]. After epithelialization, spontaneous healing is rare, and surgical correction is typically required [7].

Given this risk, early intervention within 24–48 h postextraction is critical. This window offers the best opportunity to achieve primary closure before epithelialization begins. Delayed management is associated with increased risk of sinus infection and the need for more invasive corrective procedures. As emphasized by Kamadjaja, proper and timely treatment of sinus involvement plays a decisive role in successful healing of persistent OAFs and should not be overlooked [16].

Methodologies for the treatment of OAC have evolved, focusing on both immediate closure of the communication and long-term prevention of recurrence. Methods such as the use of local flaps, bone grafts, and biomaterials have been widely documented. Various minimally invasive techniques for closing OACs include the use of PRF, Bichat's fat pad, and bone grafts. However, the utilization of PRF [17] necessitates additional equipment and preoperative preparation. Moreover, there is small risk of transmitting viral hepatitis, as noted by the manufacturer [18]. Similarly, the harvesting of Bichat's fat pad [19] and bone autografts [20] involves extraction from a donor site and entail making surgical incisions, which can result in additional patient trauma.

Visscher et al. [21] developed a method to seal OACs using biodegradable polyurethane foam, which is cylindrically shaped with a diameter of approximately 5 mm and a height of about 7 mm. However, there are potential drawbacks to this technique, which include the possibility of the foam moving into the sinus if it is too small or improperly placed, potentially necessitating a repeat procedure or surgical intervention for removal. Additionally, while the foam's porous structure facilitates rapid healing, it carries the risk of developing sinusitis, particularly if it obstructs normal sinus drainage.

The minimally invasive technique introduced by Kütük et al. [22], which utilizes soft occlusal splints, bypasses the use of sutures and involves placing a splint directly onto the socket of a freshly extracted tooth, preceded by the application of chlorhexidine gel to the splint's surface. The study reported positive outcomes with this method. However, it requires significant patient compliance; failure to consistently use the splint may lead to infection of the extraction socket.

The technique we propose for closing the OAC involves achieving mucosal mobility by carefully separating it from the periosteum with a scalpel to a depth of 0.5 cm. This allows us to stretch the mucosa and bring the wound edges as close together as possible. The sutures should be placed at least 0.5 cm from the wound margins to ensure stability. If the sutures are positioned closer than 0.5 cm to the wound edge, there is a significant risk of mucosal rupture, suture failure, and detachment of the composite material, potentially leading to complications during the healing process.

The method advocated in our clinical presentations is optimally suited for perforation defects ranging from 3 to 10 mm in diameter that manifest within 24–48 h post–tooth extraction. This approach is straightforward and does not necessitate advanced surgical expertise from the dental practitioner. The meticulous curettage of the socket of the extracted tooth prior to the application of the final layer of liquid composite enhances blood clot formation, thereby facilitating the epithelialization process without complications.

Although flowable composite resin is widely used in restorative dentistry, its direct application to mucosal tissues may pose a risk of allergic reaction or irritation [23]. Resin sensitivity and contact dermatitis are documented among dental professionals [24] and patients [25] frequently exposed to acrylate-based materials. Therefore, this technique should be avoided in patients with known resin allergies.

While the presented case reports demonstrate promising short-term outcomes, the absence of large-scale, prospective studies limits the ability to draw definitive conclusions regarding the durability, recurrence rates, and comparative effectiveness of this method relative to established surgical approaches. To validate the technique's efficacy and safety, further multicenter clinical studies, involving larger patient cohorts and randomized controlled trials (RCTs), are essential. These studies should aim to assess not only anatomical closure and symptom resolution but also long-term sinus health, bone regeneration, patient-reported outcomes, and the potential for delayed complications.

Based on our clinical experience, the use of flowable composite resin may not be suitable for very large, more than 10 mm, OACs, which could require more robust surgical interventions. The application of composite over sutures is recommended primarily for patients who do not have hemophilia or other coagulopathies that impair blood clotting. It is also advised against using this technique in patients currently on anticoagulant therapy and those with hypertension, as bleeding from the extraction socket may obstruct the effective placement of the composite resin material. The prescription of nasal decongestant drops is highly recommended to prevent obstruction and ensure proper pneumatization and aeration of the nasal sinuses.

The expansion of the use of composite resin materials in dental surgery has provided new options to promote faster healing and better results. Convenience for the surgeon and patient comfort is one of the significant advantages of the technique presented. Alternatively, the proposed method for closing OACs offers a cost-effective and practical solution for routine clinical application.

## 4. Conclusion

This report has demonstrated the feasibility of using flowable composite resin for the minimally invasive closure of OACs, highlighting its effectiveness for defects ranging from 3 to 10 mm in diameter. While offering a practical alternative to more invasive surgical methods, this technique requires careful patient selection and adherence to postoperative care to prevent complications such as infection or inadequate healing. The success of this method not only provides a cost-effective solution but also enhances patient comfort and reduces the need for extensive surgical intervention. Future research and clinical trials will be crucial in evaluating the long-term efficacy and broader applicability of this innovative approach in dental surgery.

## Data Availability

All data generated or analyzed during this study are included in the published article. Additional information can be made available by the corresponding author upon reasonable request.

## References

[1] Pawlik P., Stanek A., Wyganowska-Świątkowska M., Błochowiak K. (2019). The Epidemiological Pattern of Oroantral Communication: A Retrospective Study. *European Journal of Clinical and Experimental Medicine*.

[2] Fatani B., Fatani A., Alomar A. (2020). Oro-Antral Communication and Fistula: A Review of the Literature. *Saudi Journal of Oral and Dental Research*.

[3] Safadi A., Kleinman S., Gigi D. (2020). Surgical Management of Odontogenic Cysts Involving the Maxillary Sinus: a Retrospective Study. *Journal of Cranio-Maxillofacial Surgery*.

[4] Faé D. S., Sant’Ana G. P., Mariz B. A. L. A. (2022). Actinomycotic Osteomyelitis and Myiasis in Oroantral Communication: Clinicopathological and Tomographic Findings. *International journal of odontostomatology*.

[5] Tanna N., Awal D., Eyeson J. (2019). An Unusual Case of Sinusitis-Foreign Body in the Maxillary Antrum. *Oral Surgery*.

[6] Parvini P., Obreja K., Begic A. (2019). Decision-Making in Closure of Oroantral Communication and Fistula. *International journal of implant dentistry*.

[7] Raj G., Raj M., Loh J. S. P. (2022). Pathophysiology and Clinical Presentation of Odontogenic Maxillary Sinusitis. *Dentistry Review*.

[8] Oliva S., Lorusso F., Scarano A., D’Amario M., Murmura G. (2024). The Treatment and Management of Oroantral Communications and Fistulas: A Systematic Review and Network Metanalysis. *Dentistry Journal*.

[9] Shahrour R., Shah P., Withana T., Jung J., Syed A. Z. (2021). Oroantral Communication, Its Causes, Complications, Treatments and Radiographic Features: A Pictorial Review. *Imaging Science in Dentistry*.

[10] Ding Y., Zhu Z., Zhang X., Wang J. (2024). Novel Functional Dressing Materials for Intraoral Wound Care. *Advanced Healthcare Materials*.

[11] Nagori S. A., Jose A., Bhutia O., Roychoudhury A. (2015). A Case of Oro-Antral Communication Closed by Autotransplantation of Third Molar. *Journal of Maxillofacial and Oral Surgery*.

[12] Bereczki-Temistocle D. L., Gurzu S., Jung I. (2022). Selecting the Best Surgical Treatment Methods in Oro-Antral Communications. *International Journal of Environmental Research and Public Health*.

[13] Galli M., De Soccio G., Cialente F. (2020). Chronic Maxillary Sinusitis of Dental Origin and Oroantral Fistula: The Results of Combined Surgical Approach in an Italian University Hospital. *Bosnian Journal of Basic Medical Sciences*.

[14] Verma R. R., Verma R. (2022). Oro-Antral Fistulas and Their Management: Our Experience. *Indian Journal of Otolaryngology and Head & Neck Surgery*.

[15] Elwany N., Elwany S., Elwany S. (2024). Odontogenic Maxillary Sinusitis. *Current Rhinology*.

[16] Kamadjaja D. (2008). The Role of Proper Treatment of Maxillary Sinusitis in the Healing of Persistent Oroantral Fistula. *Dental Journal*.

[17] Adamska P., Pylińska-Dąbrowska D., Stasiak M. (2024). Treatment of Odontogenic Maxillary Sinusitis With the Use of Growth Factors in Advanced Platelet-Rich Fibrin for Immediate Closure of Oro-Antral Communication: A Case Report. *International Journal of Molecular Sciences*.

[18] Visscher S. H., van Minnen B., Bos R. R. M. (2016). Biodegradable Polyurethane for Closure of Oroantral Communications: Experimental and Clinical Evaluation.

[19] Azzouzi A., Hallab L., Chbicheb S. (2022). Diagnosis and Management of Oro-Antral Fistula: Case Series and Review. *International Journal of Surgery Case Reports*.

[20] Sim Y.-S., Jung S., Park H.-J., Kook M.-S. (2020). Closure of an Oroantral Fistula With a Bone Fragment During Surgical Extraction of an Impacted Maxillary Second Premolar: A Case Report. *Imaging Science in Dentistry*.

[21] Visscher S. H., van Minnen B., Bos R. R. M. (2010). Closure of Oroantral Communications Using Biodegradable Polyurethane Foam: A Feasibility Study. *Journal of Oral and Maxillofacial Surgery*.

[22] Kütük N., Demirbaş A. E., Yilmaz Asan C., Baş B., Alkan A. (2017). Nonsurgical Closure of Oroantral Communications Using Occlusal Splints. *Cumhuriyet Dental Journal*.

[23] Peskersoy C., Oguzhan A., Gurlek O. (2022). The Effect of Flowable Composite Resins on Periodontal Health, Cytokine Levels, and Immunoglobulins. *BioMed Research International*.

[24] Syed M., Chopra R., Sachdev V. (2015). Allergic Reactions to Dental Materials-A Systematic Review. *Journal of Clinical and Diagnostic Research: Journal of Clinical and Diagnostic Research*.

[25] Fletcher R., Harrison W., Crighton A. (2022). Dental Material Allergies and Oral Soft Tissue Reactions. *British Dental Journal*.

